# Directly measuring mean and variance of infinite-spectrum observables such as the photon orbital angular momentum

**DOI:** 10.1038/ncomms9606

**Published:** 2015-10-19

**Authors:** Bruno Piccirillo, Sergei Slussarenko, Lorenzo Marrucci, Enrico Santamato

**Affiliations:** 1 Dipartimento di Fisica, Università di Napoli Federico II, Compl. University di Monte S. Angelo, Napoli 80126, Italy; 2 Centre for Quantum Dynamics and Centre for Quantum Computation and Communication Technology, Griffith University, Brisbane, Queensland 4111, Australia; 3 CNR-SPIN, Compl. University di Monte S. Angelo, Napoli 80126, Italy

## Abstract

The standard method for experimentally determining the probability distribution of an observable in quantum mechanics is the measurement of the observable spectrum. However, for infinite-dimensional degrees of freedom, this approach would require ideally infinite or, more realistically, a very large number of measurements. Here we consider an alternative method which can yield the mean and variance of an observable of an infinite-dimensional system by measuring only a two-dimensional pointer weakly coupled with the system. In our demonstrative implementation, we determine both the mean and the variance of the orbital angular momentum of a light beam without acquiring the entire spectrum, but measuring the Stokes parameters of the optical polarization (acting as pointer), after the beam has suffered a suitable spin–orbit weak interaction. This example can provide a paradigm for a new class of useful weak quantum measurements.

## Abstract (web summary)

The more degrees of freedom a quantum observable has, the more complicated it is to measure its probability distribution. Here, the authors deduce the mean and variance of an infinite-dimensional variable, the orbital angular momentum of light, from a two-dimensional one: spin angular momentum.

## Graphical abstract

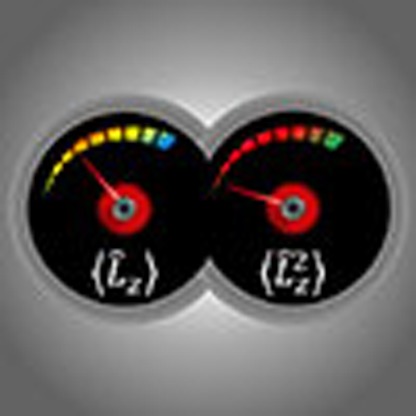

In the canonical approach to quantum measurement, a full experimental characterization of an observable *O*, for a physical system prepared in a given quantum state 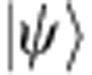, requires determining the probability distribution of its entire spectrum of eigenvalues by performing many repeated projective measurements. The statistical moments of the observable, including its mean and variance, are then also determined. For infinite-spectrum observables, however, such scheme entails the considerable overhead of having to measure a very large (ideally infinite) number of probabilities. On the other hand, one is often not really interested in such a full experimental analysis: the mean and variance of the observable *O*, although generally insufficient for fully reconstructing its spectral distribution, may provide very important information about the system and its state 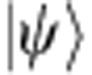. Hence, a problem of general interest in quantum theory is that of directly measuring the mean and variance of a given observable *O*, without passing through the determination of its full spectral distribution.

A noteworthy example is the long-standing problem of measuring the angular momentum of light^1^. This quantity and the ensuing rotational effects induced in matter, for example, by the circular polarization of light or by the optical ray-torsion^2^,^3^, have recently gained an increasing interest. These two cases correspond also to the natural subdivision of the angular momentum of a (paraxial) light beam into a spin part (SAM), associated with the polarization degree of freedom, and an orbital part (OAM), associated with the phase front structure^2^,^4^,^5^. The photon OAM along the propagation axis *z* can be defined as the quantum average of the operator 
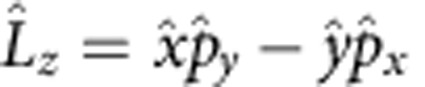
 in a given state of radiation, where 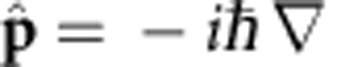 is the photon momentum operator and 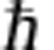 is the reduced Planck constant^5^. Consistently, the fraction of the total intensity belonging to the helical transverse modes characterized by the phase factor 
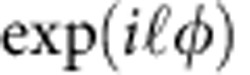
, where *φ* is the azimuthal coordinate around the *z* axis, can be identified with the probability of obtaining 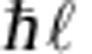 in an ideal projective measurement of the OAM of the photon in that state^4^,^6^. The OAM is defined over an infinite-dimensional Hilbert space and has a discrete infinite spectrum, sometimes named as ‘spiral spectrum'. Most existing methods for measuring such spiral spectrum are based on filtering or splitting the wave field according to the OAM eigenmodes, that is, using mode selectors^7^,^8^,^9^,^10^ or mode sorters^11^,^12^,^13^,^14^,^15^,^16^,^17^. However, in all those cases in which the spiral spectrum is not confined *a priori* to a sufficiently small range, this projective scheme for determining the whole spectrum requires a very large number of measurements. On the other hand, one is often only interested in determining the average OAM and, possibly, its variance, without passing through the determination of the entire spectrum. OAM mean and variance may, for example, provide partial but important information about the source of the radiation field, or about angular momentum conservation in radiation–matter interactions^18^,^19^.

To tackle the problem we posed, we may exploit the theory of quantum weak measurements (WMs). This theory, developed by Aharonov *et al*.^20^, has recently gained a great deal of interest due to the possibility of extracting information from a measurement process while disturbing the state of the observed system as little as possible. In particular, much attention was focused on the more specific concept of ‘weak values' (WV), which arise in a WM when the measurement is combined with post selection of the observed system on a given final state. WMs (and WVs) have proved to be a powerful tool for addressing fundamental questions in quantum mechanics, such as the direct measurement of the wave function of a particle^21^,^22^,^23^, as well as for technical applications, such as enhanced precision measurements^24^,^25^ or the active control of quantum systems^26^,^27^.

WMs are based on the ancilla measurement scheme which generalizes the von Neuman protocol^28^ and comprises two essential stages: ‘premeasurement', which consists in the interaction process between the object–system and the measurement device, also called pointer or ancilla, and ‘read-out', which consists in a projective (strong) measurement on the ancilla. The disturbance on the object–system is reduced by weakening the interaction underlying the premeasurement. When the premeasurement is also followed by another projective measurement that post-selects a particular final state of the object–system (besides the ancilla), one obtains the above mentioned WVs. In our case, however, WVs play no role. We will benefit instead from a thus far unexploited feature of WMs that consists in their capability to obtain the mean (or expected) value of an object–system variable 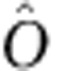 by measuring the mean value of an ancilla variable 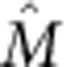, after a suitable weak interaction between the two (taking place during the premeasurement) and without post selecting the object–system on a given final state (case named as ‘standard' weak measurements, SWMs)^29^,^30^. Such a ‘weak mean' can be proved to be equal to the quantum expectation value of the observable 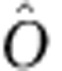 determined by strong measurements^28^. Though apparently the measurement problem in this way is just shifted from the object to the ancilla, this is not without advantages. In general, the ancilla's observable can be more easily measured than the object's one, and it can be also simpler in structure, for example by having a smaller Hilbert space dimensionality. Moreover, by exploiting the same weak interaction process it is possible to extract multiple elements of information (limited by ancilla dimensionality) about the object–system by considering different projective measurements on the ancilla.

In the demonstrative implementation we present here, the object's observable is the photon OAM, which is infinite dimensional, while the ancilla is the photon polarization, which defines a two-dimensional Hilbert space and is hence much easier to be measured. Moreover, as we shall see, it is possible extracting not only the mean but also the second order moment (and hence the variance) of the object-observable statistical distribution just by selecting two suitable observables of the ancilla. We are not aware of prior demonstrations of this kind of applications of the WM concept, which in our opinion can have wide applicability in different areas of quantum physics.

## Results

### Concept of the experimental method

**Figure 1:**
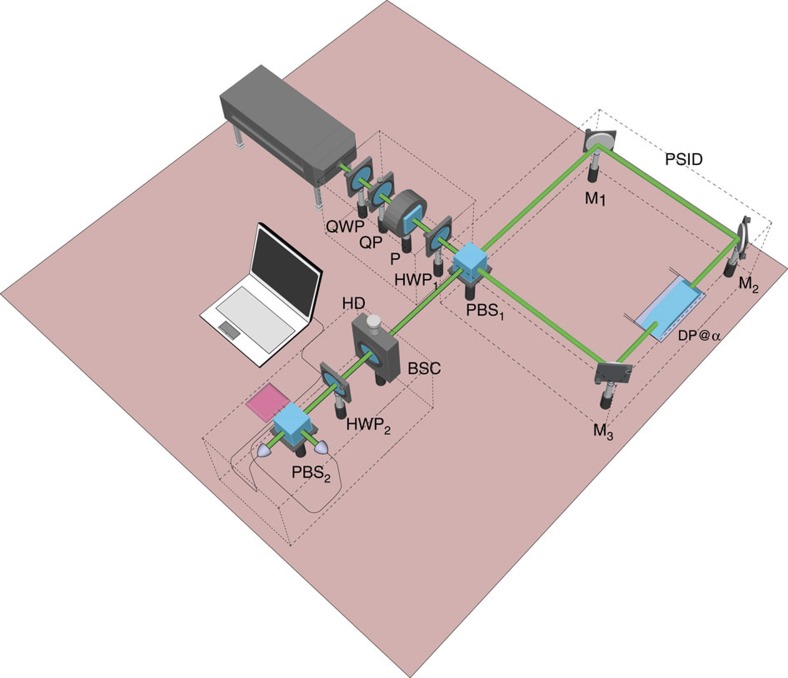
Schematic of the setup for measuring the first and second moments of the OAM probability distribution carried by a paraxial light beam. An initially Gaussian beam is converted into an OAM superposition state using a quarter waveplate (QWP) and a *q*-plate (QP). A polarizer (P) and a half waveplate (HWP_1_) are then used to prepare the polarization in a linear state oriented at 45° with respect to the axes of the polarizing beam splitter (PBS_1_). This PBS_1_ is the input/output port of a polarizing Sagnac interferometer (PSID)^31^,^36^, whose path is closed by mirrors M_1_, M_2_ and M_3_, that contains the Dove prism used to rotate the two counterpropagating beams with respect to each other. At the output of the PSID, a Babinet–Soleil compensator (BSC) is used to adjust the relative phase-shift δ. The last stage is the balanced polarizing homodyne detector HD, with the axes rotated by 45° with respect to the PSID axes through the half waveplate HWP_2_.

In our method, the object–system is represented by the OAM carried by an arbitrarily prepared paraxial beam and the ancilla or meter by its SAM. In the premeasurement, OAM and SAM are made to interact through a Sagnac Polarizing Interferometer containing a Dove prism (PSID)^31^ as shown in Fig. 1. Essentially the input beam is first divided by the polarizing beam splitter (PBS_1_) into two copies orthogonally polarized along the horizontal *H* and vertical *V* directions, respectively. These copies, through the Dove prism, are rotated with respect to each other by a small angle around the propagation axis. The base of the Dove prism is located in a plane tilted by the angle *α* with respect to the horizontal plane containing the interferometer. The intersection axis between such planes makes the rotation axis of the PSID. Consequently, the beams propagating in opposite directions along the arms of the PSID suffer opposite azimuthal angular shifts of 2*α*, leading to their relative rotation of 4*α*. This provides the premeasurement stage. After exiting the PSID, the *H*- and *V*-polarized beams are then superimposed in the final homodyne detection (or read-out) stage, as shown in Fig. 1. The total system OAM+SAM of a light field is assumed to be prepared in a separable state represented by the initial density matrix 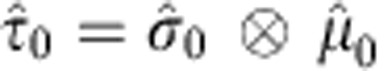, where 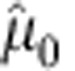 is the initial density matrix of the SAM (or polarization), playing the role of the ancilla or meter, and 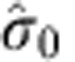 is the initial density matrix of the OAM, playing in turn the role of the object. The SAM initial state is always assumed to be pure, while the initial OAM state may be either pure or mixed. For the sake of simplicity, in the experimental demonstration that follows, we assumed the OAM initial state to be a pure (that is coherent) finite or infinite superposition of helical modes, so that 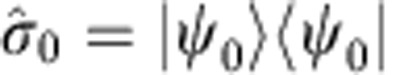, while the SAM initial state was chosen to be the antidiagonal polarization state 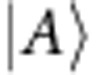 so that 
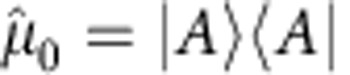
. The premeasurement is described by the evolution operator


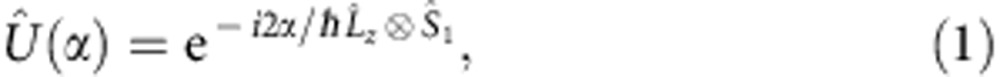


where 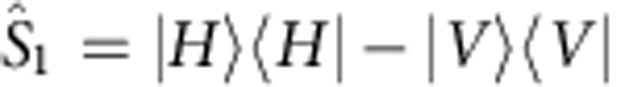 is the first Stokes parameter observable, 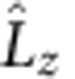 is the OAM operator with respect to the PSID axis and 2*α* is the effective coupling constant. 
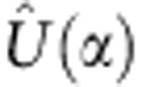
 is able to distinguish between the different helical modes 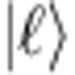, since 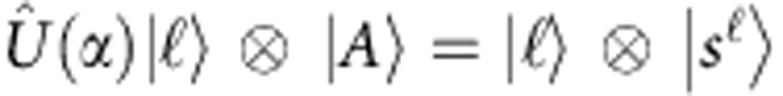, with the SAM state 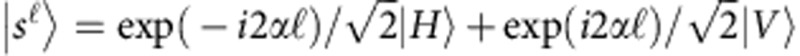 acting as marker for 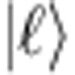. The premeasurement correlates the polarization components with the orientation of their transverse azimuthal phase distribution by rotating them in opposite directions, yielding a SAM–OAM entangled state. The object–device interaction is weakened by decreasing the Dove prism rotation angle *α*, which amounts to decreasing the effective coupling constant 2*α*. Noticing that in the initial state, 
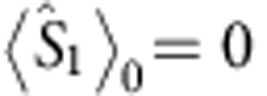
 and 
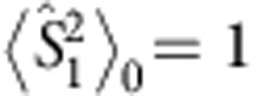
, disregarding terms of order *α*^3^ and higher, the final OAM unconditional density matrix reads


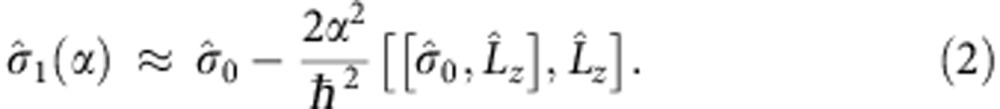


In the same approximation, the final unconditional SAM matrix density reads


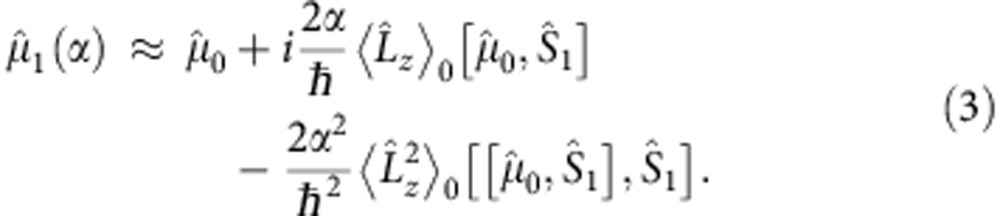


Generalizing the von Neumann protocol, the premeasurement device, PSID, can be used to translate the measurements of the mean and variance of the OAM of the input beam into the measurements of the means of two suitable polarization ‘pointers', specifically the polarization Stokes observables of the output beam. In detail,


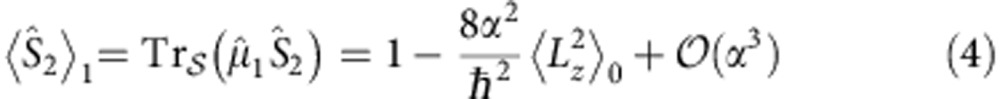



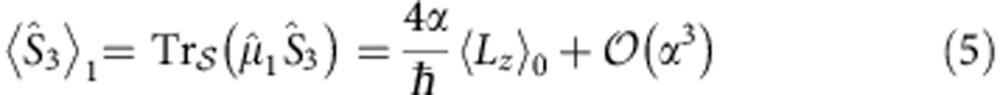


where 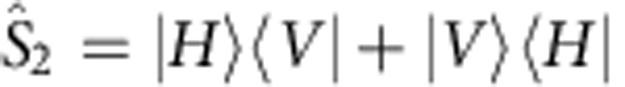 and 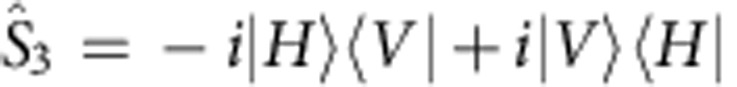 are the second and third Stokes parameters, respectively. The amount of information returned by SWMs when applied to a single system is almost vanishing, since the average ‘pointer deflection' is much less than the pointer uncertainty. Consequently, a reliable estimation of the mean of the object-observable can be obtained only by performing the measurements on each member of a sufficiently large ensemble of systems all prepared (preselected) in the same state. Therefore 
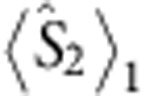
 and 
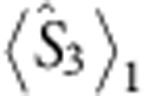
 have been measured by the homodyne detection stage HD (Fig. 1 and Fig. 5), which makes the scheme suitable for both continuous wave and photon-counting operation (see the section ‘The homodyne detection method' in Methods). The optical powers *P*_+_ and *P*_−_ at the output ports of PBS_2_ are electronically processed to provide the total power *P*_0_=*P*_+_+*P*_−_ and the difference Δ*P*=*P*_+_−*P*_−_, so that


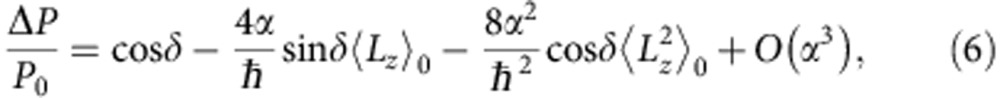


where *δ* is the phase retardation between the *H*- and *V*-polarized photons exiting the PSID and can be tuned through the Babinet–Soleil compensator. From equation (6), we obtain 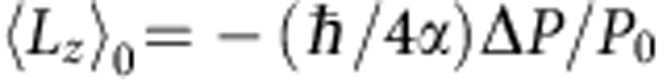 for 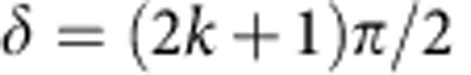, and 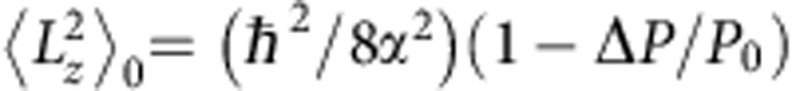 for *δ*=2*k*π, with *k* being an integer. The preselected value of *α* is set through the goniometer *G*(±0.008°) and an accurate estimate for its value was then obtained by the calibration procedure. Zeroing and calibration of the apparatus were carried out by adopting as standard input the OAM imparted by perfectly tuned *q*-plates^9^,^32^, for several values of *q* (with *q*≤8), onto the high-quality TEM_00_ mode generated by a single-mode continuous wave laser (wavelength *λ*=532 nm). The selection criterium for *α* is a critical point of the method. In fact, it sets an upper limit to the maximum value of 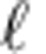 that can be measured within a specified accuracy (upper cutoff) and controls the signal-to-noise ratio. For instance, to set the cutoff 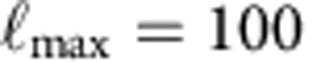, maintaining within 1% the theoretical accuracy in the measurement of both 
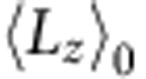
 and 
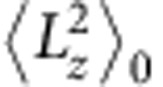
, an *α*≈0.05° is to be selected. Since, the signal returning 
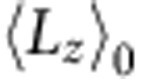
 is proportional to *α* while that returning 
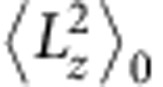
 is proportional to *α*^2^, to accurately discern the eigenstate 
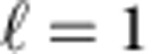
, the noise in the signals must be lower than ≈10^−5^. This requires a very precise control of the optical path within the PSID, which reflects into a good control of the noise in the phase retardation *δ*. Our experiment was carried out with *α*=(0.85±0.01)°, corresponding to 
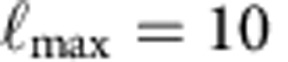
 and a maximum acceptable noise in the signals of ≈10^−3^.

### Experimental demonstration

To validate our method, after calibration on OAM eigenstates, we measured the moments 
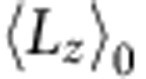
 and 
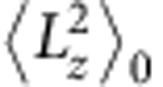
 of light beams containing different OAM superpositions. These beams were all obtained from a Gaussian TEM_00_ laser beam (VERDI LASER, @*λ*=532 nm, continuous wave operation) using *q*-plates (QP) with different charges *q*, while varying the following parameters: (i) the polarization before the QP, (ii) the QP birefringent retardation (that is, the QP ‘tuning') and (iii) the relative alignment of the beam axis and the QP centre. After passing through the QP, the beam polarization is reset to the 45° linear polarization needed for erasing the SAM–OAM correlation established by the QP due to its intrinsic birefringence and prepared for the subsequent OAM measurement.

When the QP is centred on the beam axis, for each *q* the generated OAM superpositions involve just three OAM eigenstates, that is, 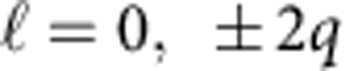. The relative weight of these three states in the superposition can be controlled using the polarization input helicity *s*_3_ before the QP and the QP phase retardation *θ*, the latter being controlled by the QP voltage V. The theoretical expressions for the first and second moments of the resulting OAM distribution can be easily calculated and are given by


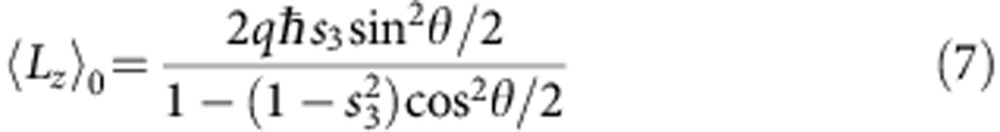


and


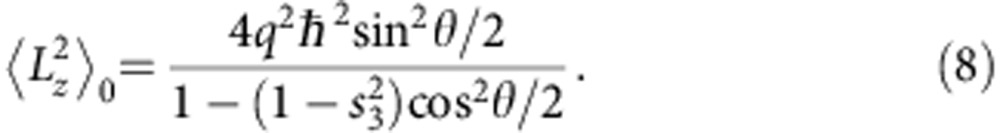


**Figure 2:**
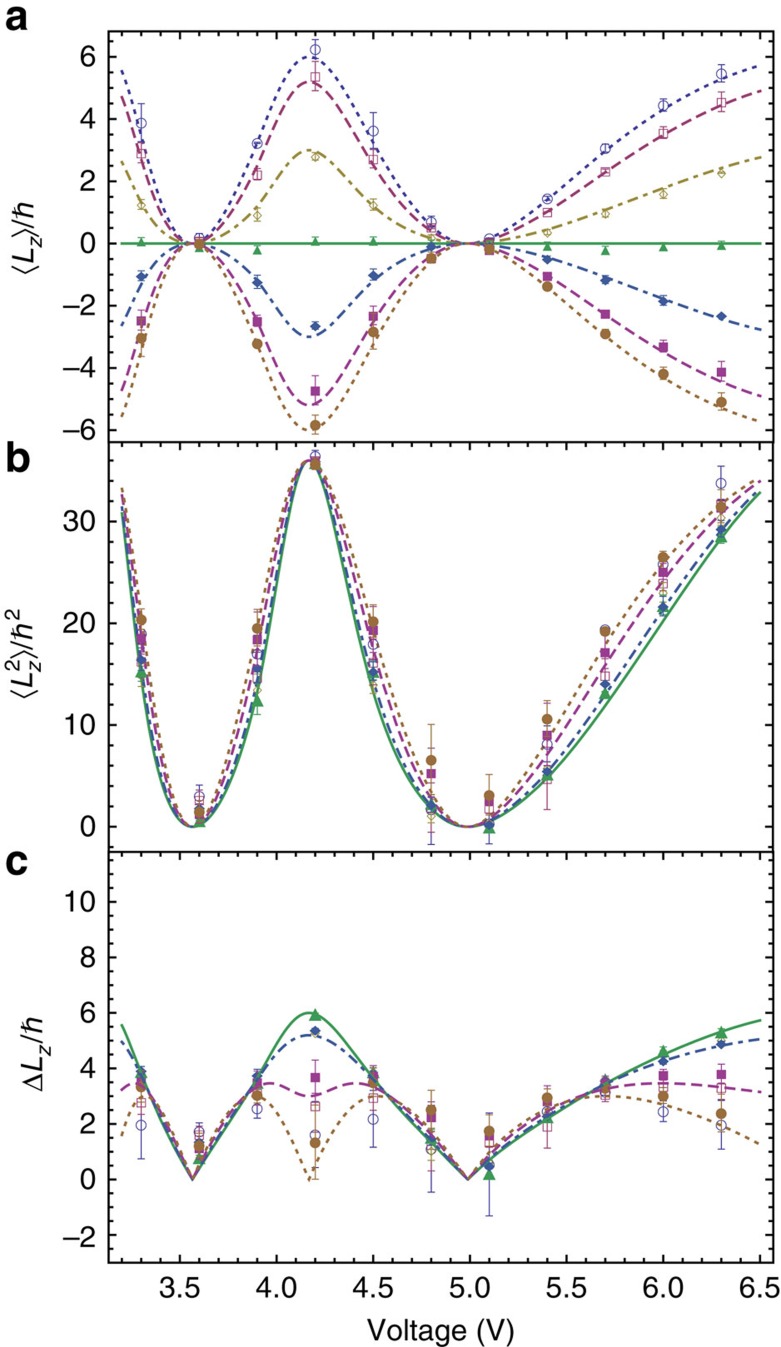
Measurement of the OAM moments of a superposition of 
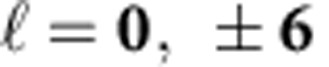
 with variable coefficients. Behaviour of the first (**a**) and second (**b**) moment and of the root mean square deviation (**c**) of the OAM distribution of the beam prepared using a *q*-plate, as a function of the *q*-plate tuning voltage controlling its retardation *θ* and for different values of the polarization helicity *s*_3_ before the *q*-plate. The two parameters *θ* and *s*_3_, together with the q-plate charge *q*, define the specific OAM superposition of states having 
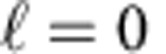
 and 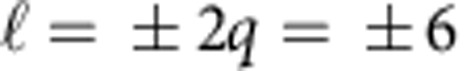. Experimental data points for 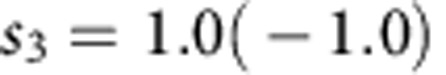 are represented by ○ (○), for 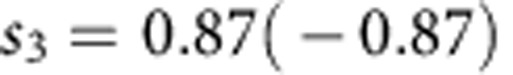 by □ (▪), for *s*_3_=0.50(−0.50) by ◊ (♦) and for *s*_3_=0 by ▴. Error bars represent standard deviations due to misalignment errors, estimated by repeating the experiments after realignment. The curves give the corresponding theoretical predictions calculated from equation (7) for *s*_3_=±1 (dotted curves), *s*_3_=±0.87 (dashed curves), *s*_3_=±0.50 (dot-dashed curves) and *s*_3_=0 (solid curve).

**Figure 3:**
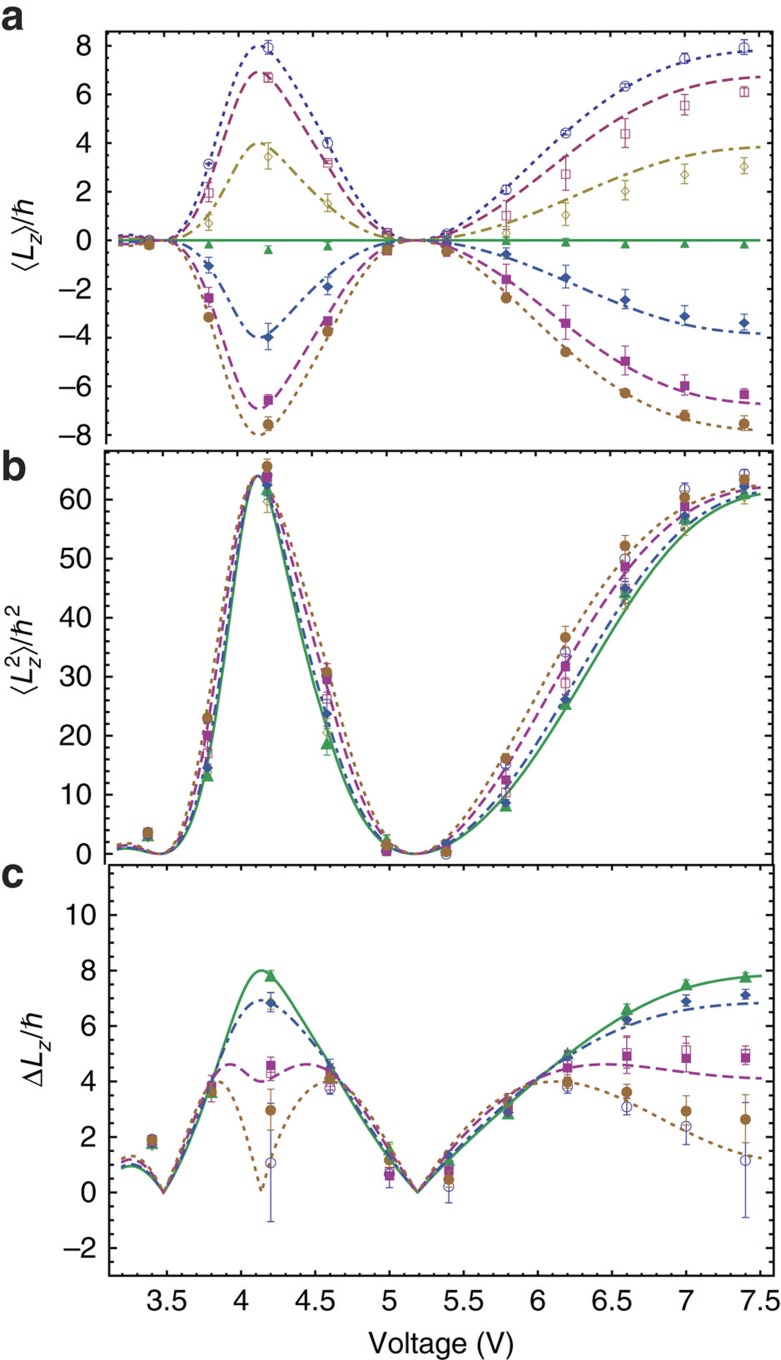
Measurement of the OAM moments of a superposition of 
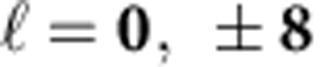
 with variable coefficients. Behaviour of the first (**a**) and second (**b**) moment and of the root mean square deviation (**c**) of the OAM distribution of the beam prepared using a *q*-plate, as a function of the *q*-plate tuning voltage controlling its retardation *θ* and for different values of the polarization helicity *s*_3_ before the *q*-plate. The two parameters *θ* and *s*_3_, together with the *q*-plate charge *q*, define the specific OAM superposition of states having 
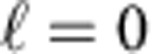
 and 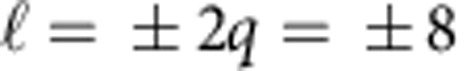. Experimental data points for *s*_3_=1.0(−1.0) are represented by ○ (○), for *s*_3_=0.87(−0.87) by □ (▪), for *s*_3_=0.50(−0.50) by ◊ (♦) and for *s*_3_=0 by ▴. Error bars represent standard deviations due to misalignment errors, estimated by repeating the experiments after realignment. The curves give the corresponding theoretical predictions calculated from equation (7) for *s*_3_=±1 (dotted curves), *s*_3_=±0.87 (dashed curves), *s*_3_=±0.50 (dot-dashed curves) and *s*_3_=0 (solid curve).

The (device-dependent) tuning function *θ*(V) is obtained from an independent characterization of the QP birefringent retardation^33^. The results of our measurements on these OAM superposition states are shown in Figs 2 and 3, together with the theoretical predictions from equations (7) and (8). The agreement is clearly very good, particularly considering that there are no adjustable parameters in the theory. However, the calibration of the setup is not entirely independent from these data, as pure OAM eigenstates correspond to the first two opposite maxima obtained in the outermost curves at a voltage of about 4.2 V (for which *θ*=*π* and the QP is tuned). In particular, as mentioned, the precise value of the Dove prism angle *α* with respect to the PSID plane was estimated by matching the measured values of mean OAM to the theoretical ones in these specific cases.

**Figure 4:**
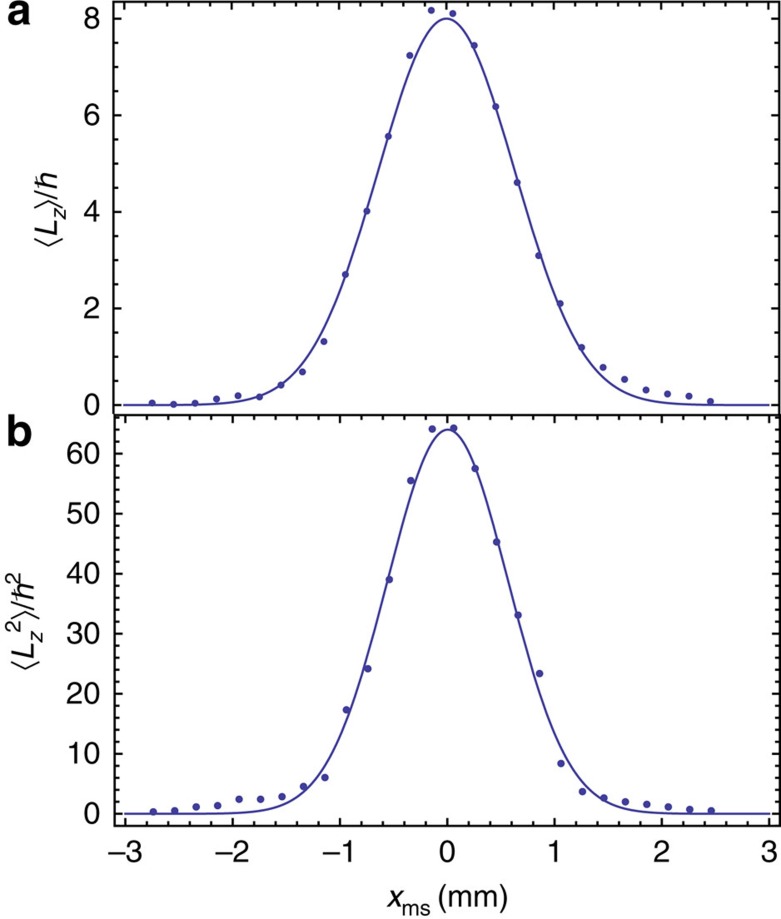
Measurement of the OAM moments of a beam having a broad spiral spectrum. Behaviour of the measured first (**a**) and second (**b**) moment of the OAM distribution of the beam as a function of the misalignment distance *x*_ms_ between the centre of the beam and the centre of the vortex singularity introduced by the tuned *q*-plate with *q*=4. The theoretical predictions are represented by the solid curves. The typical experimental uncertainties on the first and second moment data points are 
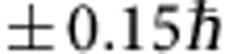
 and 
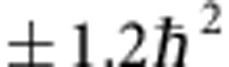
, respectively.

To test the validity of our method in a more complex situation, we then measured the first and second moments of the OAM distribution of a beam generated by means of a perfectly tuned *q*-plate (*θ*=*π*) with *q*=4, for circular polarization input (*s*_3_=1.0), whose centre is translated off of the beam axis by a variable distance *x*_ms_. In this situation, the input beam in the measurement setup is similar to a Gaussian beam with an added vortex located at a distance *x*_ms_ from the beam axis. This is not an eigenstate of OAM but is a superposition of many different values of 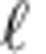. In such case, the theoretical predictions for the first two moments of the OAM distribution can be shown to be the following: 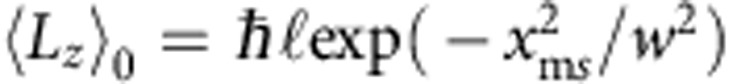 and 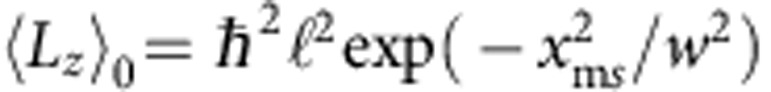, with 
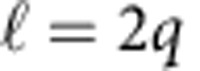
 and ω the beam waist in the plane of the *q*-plate. We stress that these results are based on setting the origin of the coordinate system in the beam centre (and not in the vortex centre). Experimentally, this is fixed by aligning the beam axis with the rotation axis of the Dove prisms in the PSID. The experimental results for this test are reported in Fig. 4, where they are compared with the above theoretical predictions, showing again a very satisfactory agreement.

## Discussion

In this paper, we have demonstrated that a possible approach to overcome the practical difficulties that arise when measuring the probability distribution of an infinite-spectrum quantum observable is provided by weak measurements without post selection or ‘standard' weak measurements. We have proposed to exploit the capability of standard weak measurements to shift the problem of getting the statistics from the object to the ancilla, to acquire multiple elements of information about a complex object system via measurements performed on a much simpler ancilla. In particular, we applied this method to measuring both the mean and the variance of the OAM distribution of a general paraxial optical beam by doing only standard polarimetry. Immediately after the premeasurement, which entangles weakly the OAM and polarization of the input beam state, without introducing any post selection on a specific transverse OAM mode, the required moments of the input OAM distribution turn out to be proportional to the output polarization Stokes parameters and can therefore be easily measured through a polarizing homodyne scheme. Our demonstrative result is important in itself, as it is the first measurement of mean and variance of the optical OAM that does not pass through the acquisition of the full spiral (OAM) spectrum. Such a scheme could be of great help in experimental studies of OAM exchange in light–matter interaction or to weaken the usual alignment requirements typical of OAM-based free-space communication^12^,^34^,^35^. We notice that our demonstration is based on a purely classical optics experiment (not differently from previous WM demonstrations, for example, refs 24, 21), which could also be discussed entirely in classical terms, not requiring WM or even quantum concepts. However, the quantum interpretation we provide of our experiment is much more general and also applicable to systems which are entirely nonclassical. Schemes of weak measurements without post-selection modelled after our example are expected to find application in many other quantum metrological problems, for example in which the object observable cannot be easily accessed directly or in which the interaction with the measuring device is constrained.

## Methods

### The premeasurement

In the measurement, the observer couples the uknown OAM input state (observed system) to SAM (pointer or ancilla) through the PSID. This coupling is represented by the unitary operator 
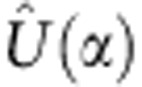
 in equation (1). Let the OAM input state be represented by the initial density matrix 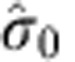 and manipulate the photon polarization so that the SAM input state is 
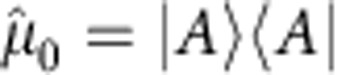
, that is, antidiagonal with respect to the PSID axes. The initial state of the total system OAM+SAM is separable and is therefore represented by the density matrix 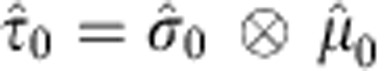. The SAM–OAM entangled state arising from the premeasurement is


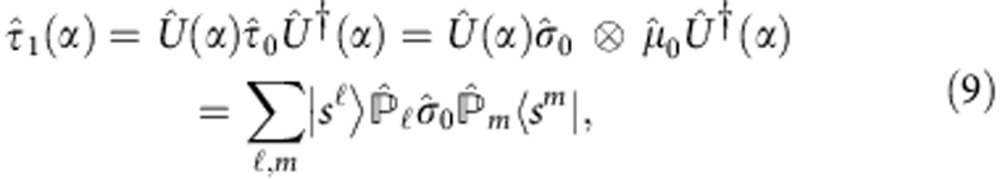


where 
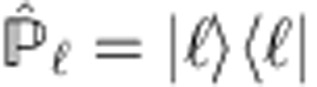
 is the projector operator along the 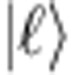 state and 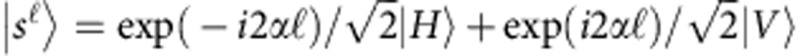 is the SAM state marking the OAM state 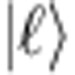. The separate states 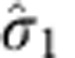 for the system and 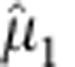 for the ancilla, after the premeasurement, are obtained by taking the partial trace over the non-interesting degrees of freedom. Therefore, in the weak coupling regime, to the second order in *α*, the final OAM state unconditional density matrix 
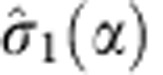
, as reported in equation (2), is given by


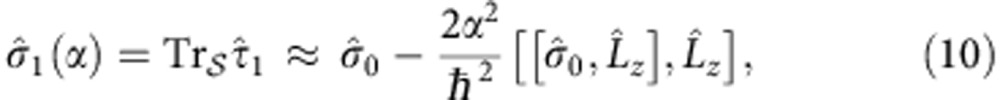


where 
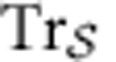
 denotes the partial trace over the SAM degrees of freedom. The matrix elements of 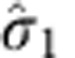 in the 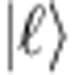-basis are


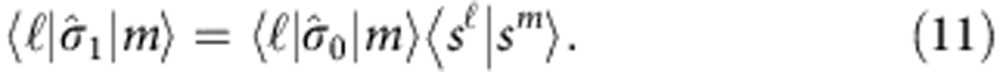


For a weak measurement there is almost total overlap between different pointer states 
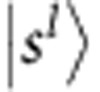
 (while for an ideal strong measurement the overlap tends to zero). Specifically the overlap of the final polarization states is


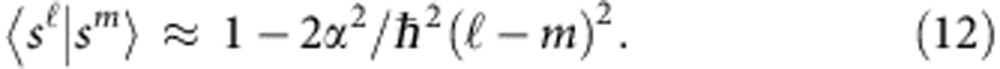


This implies that the correction to the initial OAM density matrix is even of second order in *α*. Analogously, the unconditional density matrix 
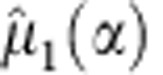
 of the final SAM state is obtained from 
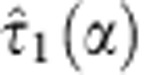
 as


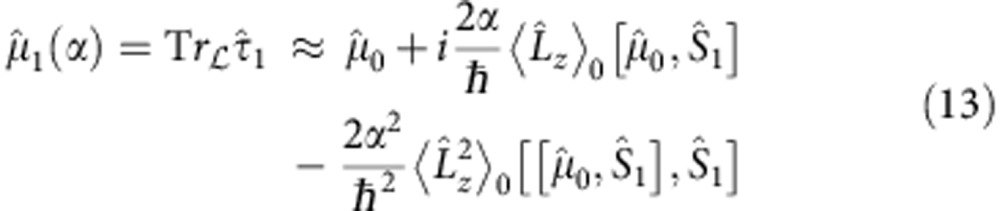


where 
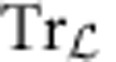
 denotes the partial trace over the OAM degrees of freedom. Form the density matrix 
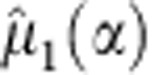
, the expectation values of all the pointer variables after the premeasurement can be calculated. In particular, the expectation value of 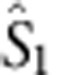 is conserved in the measurement, while the expectation values of 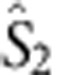 and 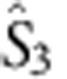 in the final state of the ancilla are given by equations (4) and (5), which are related to 
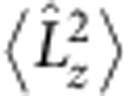
 and 
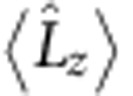
, respectively.

In conclusion, in weak coupling regime, the measurements of both the mean and variance of the photon OAM are translated by the premeasurement into the measurements of two distinct variables of the same pointer or ancilla, 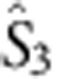 and 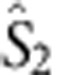 respectively. However, 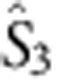 yields 
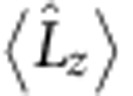
 to the first order in the coupling constant 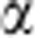 and 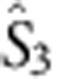 yields 
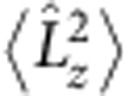
 to the second order in α.

### The homodyne detection method

**Figure 5:**
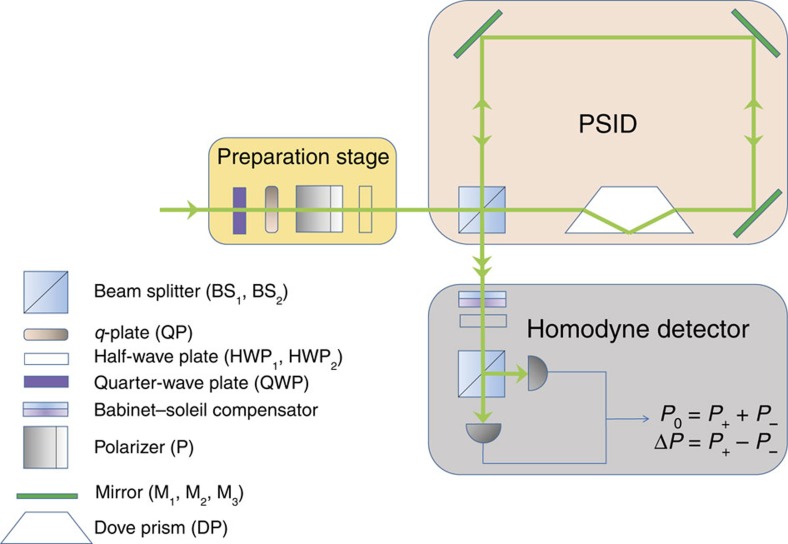
Block diagram of the setup for measuring the first and second moments of the OAM probability distribution carried by a paraxial light beam. An initially Gaussian beam is converted into an OAM superposition state using a quarter waveplate (QWP) and a *q*-plate (QP). A polarizer (P) and a half waveplate HWP_1_ are then used to prepare the polarization in a linear state oriented at 45° with respect to the axes of the polarizing beam splitter PBS_1_. This PBS_1_ is the input/output port of a polarizing Sagnac interferometer (PSID)^31^,^36^, whose path is closed by mirrors M_1_, M_2_ and M_3_, that contains the Dove prism used to rotate the two counterpropagating beams with respect to each other. At the output of the PSID, a Babinet–Soleil compensator (BSC) is used to adjust the relative phase shift *δ*. The last stage is the balanced polarizing homodyne detector HD, with the axes rotated by 45° with respect to the PSID axes through the half-waveplate HWP_2_ (Fig. 1).

The Stokes parameters of the beam after the premeasurement, 
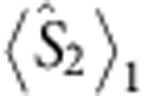
 and 
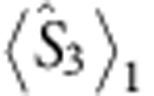
, have been measured by the homodyne detection stage HD (Fig. 5). The *H*- and *V*-polarized photons exiting the PSID pass through a *δ* retardation waveplate and are then made interfere through the polarizing beam splitter PBS_2_. In this way, the optical powers *P*_+_ and *P*_−_ at the output ports of PBS_2_ are respectively proportional to the probabilities that an input photon in the initial polarization state 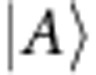 is dragged by the PSID towards the final polarization states 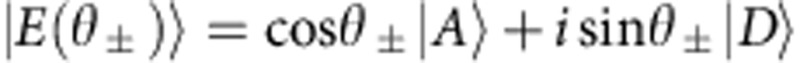, *θ*_+_=*δ* and *θ*_−_=*δ*–*π*. *δ* is the phase retardation between the *H*- and *V*-polarized photons exiting the PSID and can be tuned through the Babinet–Soleil compensator. A straightforward calculation shows that


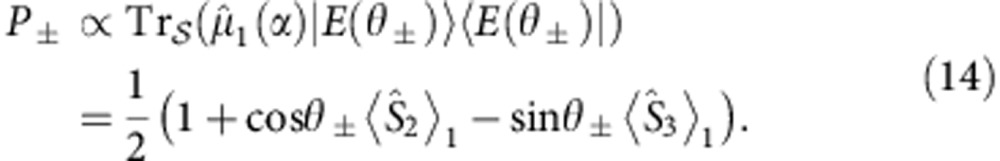


*P*_+_ and *P*_−_ are then electronically processed to provide the total power *P*_0_=*P*_+_+*P*_−_ and the difference Δ*P*=*P*_+_−*P*_−_, involved in equation (6). In practice, in the experiment, *δ* was set to *π* to measure 
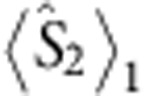
 or to *π*/2 to measure 
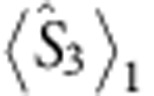
.

## Additional information

**How to cite this article:** Piccirillo, B. *et al*. Directly measuring mean and variance of infinite-spectrum observables such as the photon orbital angular momentum. *Nat. Commun*. 6:8606 doi: 10.1038/ncomms9606 (2015).
